# Supplementary material for: Clone-structured graph representations enable flexible learning and vicarious evaluation of cognitive maps
Source: Nat Commun. 2021 Apr 22;12:2392. doi: 10.1038/s41467-021-22559-5 (PMC8062558; doi:10.1038/s41467-021-22559-5)
Supplement: Supplementary file 1 — Supplementary Information [file 41467_2021_22559_MOESM1_ESM.pdf]

Supplementary Results

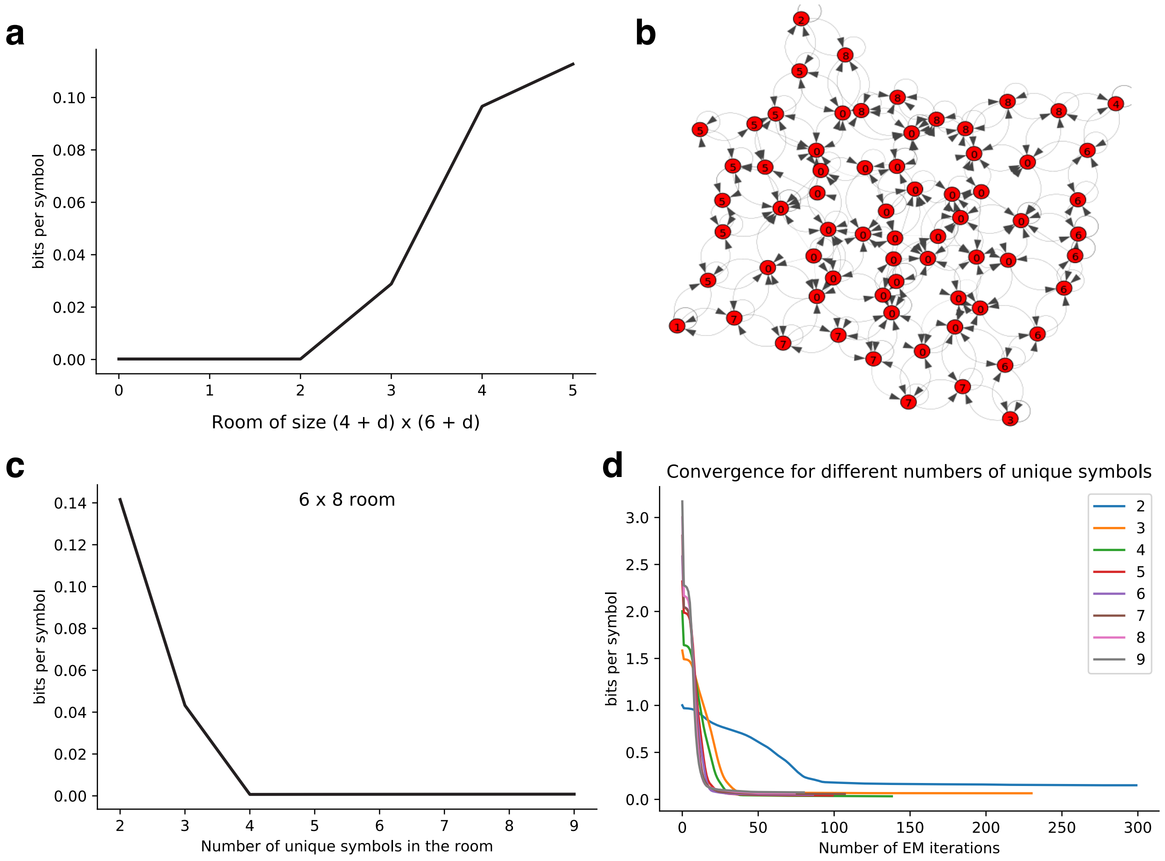

Supplementary Fig. 1: The effect of varying room size and the number of unique observations in each room. **(a)** Larger empty rooms result in increased bps. **(b)** Imperfect graph learned from a large empty room. **(c)** Fewer unique observations result in increased bps. **(d)** Fewer unique observations make learning harder.

Learning spatial representations

In order to learn the spatial representation of a room with a CSCG, we let an agent roam in a room and receive a stream of local visual cues paired with the executed actions. Note that there are two factors that complicate learning: on the one hand, the visual cues do not need to be unique to each location in the room. In fact, in an empty room, every location in the room away from the walls and the corners looks the same (see Fig. 2c). On the other hand, even though the agent's proprioception lets it know

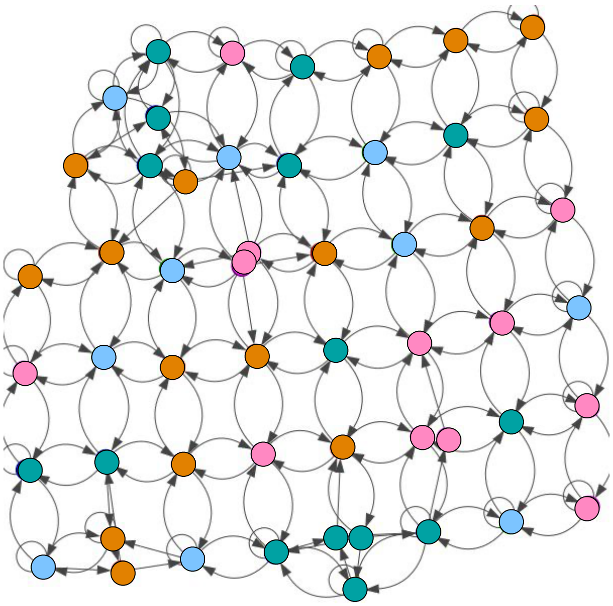

Supplementary Fig. 2: The room in Fig 2a learned without observing action sequences. CSCG was able to recover an imperfect layout of the room, showing that observing the actions help, and absence of those observations produce a degradation, but not necessarily a catastrophic failure. Smaller rooms are recovered perfectly, despite aliasing and not observing the actions.

the identifier of the executed action, there is no meaning associated to it. I.e., every time that the agent moves west, it knows it is doing the same thing, but it does not have any prior knowledge of what that thing is.

In the case of empty rooms, most of the observations received by the agent are the same (the empty observation, as it wanders through empty space) and it is hard for the agent to locate itself in the room, since only the walls and corners provide context. We have experimented with CSCGs learning in empty rooms of different sizes. We use 50000 steps[2] in a room of size $(4 + d) \times (6 + d)$, where d is a parameter controlling the room size. For rooms of size 6×8 and below, EM learning recovers exactly the structure of the room. For larger sizes, it starts to make some mistakes in its understanding of the room, slightly decreasing its predictive ability as the room grows, see **Supplementary Fig. 1a**. **Supplementary Fig. 1b** shows the learned transition matrix in graph form for a room of size 9×11. The graph looks almost perfect, but if we follow the path between observations '1' and '3', we should traverse seven observations

[2]Other parameters for this experiment are the number of clones used (70, which is theoretically enough to exactly recover even the largest room), a pseudocount of $2 \cdot 10^{-3}$ used in the EM procedure and a maximum number of EM iterations of 1000.

of type '7', whereas there are only six. The CSCG has merged two physical locations in the room (that have a large neighborhood of identical sensory cues) into the same perceived location.

Learning the structure of a room would be trivial if each observation was unique to a single room location. In that case, a CSCG with only one clone would learn the correct solution in one EM step. We experiment with different numbers of unique symbols randomly placed in a room. **Supplementary Fig. 1c** shows that in a room of size 6×8 (depicted in **Fig. 2a**) the performance degrades as the number of unique symbols decreases, with recovery being exact only when the number of unique symbols is 4 or more. The number of EM iterations required for convergence[3] is also affected, as shown in **Supplementary Fig. 1d**.

Reusing learned graphs as a schema

Using the learned transition graph as a schema assumes that the transition structure remains constant between the learned setting and the new setting, while the mapping to the observations, the emission matrix, is relearned. Relearning the emission matrix can also tolerate some imperfections in the mapping between the learned graph and the new environment it is trying to model. If the new layout is smaller than the original room, the smaller layout will still be learned well just by using parts of the original transition matrix. Here we show that learning larger layouts compared to the original layout results in a gradual degradation, of prediction accuracy.

In the original experiment, we showed that reusing the graph for a learned 6×8 room speeds up learning for a new room with identical layout but different observation mapping. We further tested this with three novel environments whose layouts differed from the original 6×8 layout – 7×9, 8×10, and 9×11. All novel layouts used 20 distinct observations, like in the original experiment. While the original 6×8 layout was learned with a prediction accuracy 0.99, the 7×9, 8×10, and 9×11 layouts were learned with prediction accuracies of 0.534, 0.423, and 0.332 respectively, showing a gradual degradation in prediction accuracy as the layout of the new room increased in size in relation to the reference layout. Note that the prediction accuracies using an imperfectly matched graph are still significantly higher than

[3]The parameters for this experiment are: 10000 recorded steps, a CSCG with 30 clones (enough for exact recovery for all the numbers of unique symbols tested), a pseudocount of 10^{-2} in the EM procedure and a maximum number of EM iterations of 1000.

the chance level accuracy of 0.05 obtained without the use of a graph.

Event-specific representations in maze elongated with novel observations

In the maze elongation experiment in Fig 5(d), the maze was elongated by repeating two observations along the horizontal arms. We performed an additional experiment to test whether the clone activity traces will be similarly preserved if the maze was elongated with a novel observation instead. Fig 3 shows the resultant clone activity traces. Similar to the original experiment the clone activity trances are preserved across this expanded section because the novel observation appears as observation noise and smoothing in CSCG ensures that the history is maintained during the noisy section.

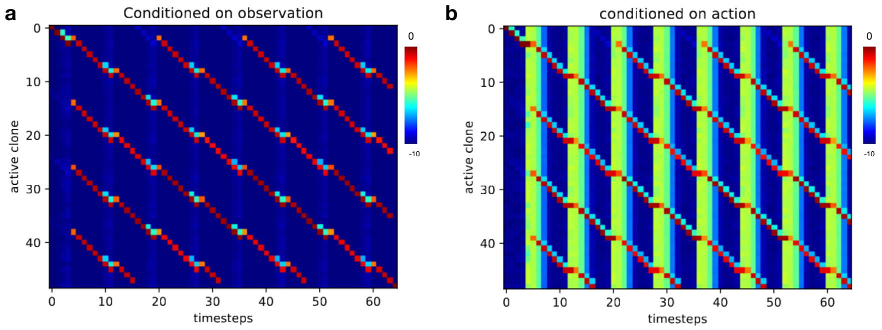

Supplementary Fig. 3: Clone activity traces for expanded maze with novel observations. The horizontal arm of the original maze in Fig 5d is expanded using novel observations instead of repeating the observations in the maze. Event-specific activity traces are preserved due to smoothing in CSCG.**(a)** CSCG activity traces conditioned on observation. **(b)** CSCG activity traces conditioned on action.

CSCG and community detection for fully observed graph

When the graph in Fig 7**a** is fully observed, with a unique observation per node, CSCGs learn the transition graph as expected. Community detection on this transition graph recovers the communities, as shown in Fig 4, demonstrating that forming SR of the transition matrix is not a necessary step for community detection.

Successor representation of the CSCG transition matrix

The CSCG model contains all the information about the sequence generating process, so it can be combined with an external policy to yield the successor representation associated to that policy. The suc-

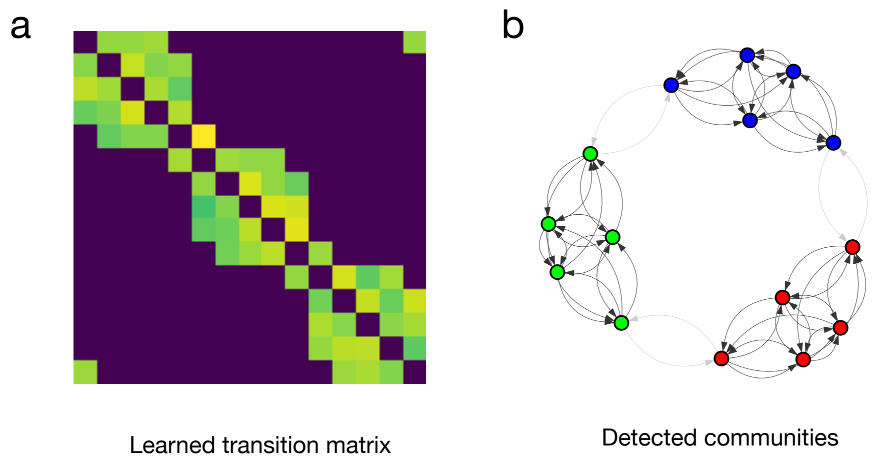

Supplementary Fig. 4: CSCG Transition matrix **(a)** and detected communities **(b)** for the case in which the graph is Fig 7**a** is fully observed with a unique observation per node. The communities are recovered correctly from the transition matrix alone, without having to form an SR matrix. The colors in the transition matrix represent probabilities, with zero being represented by purple and non-zero values being represented by shades of green to yellow.

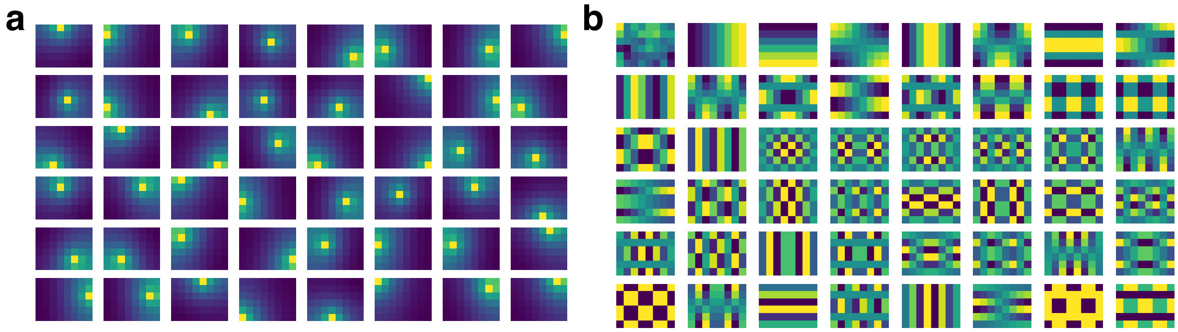

Supplementary Fig. 5: Successor representation and eigenvectors derived from the transition matrix of a CSCG. The CSCG was trained with data collected from a random walk in a rectangular room. **(a)** Successor Representations in 6×8 room. **(b)** Eigenvectors of Successor Representation.

cessor representation[25, 60] loses precise temporal information and, as a result, contains strictly less information than the CSCG. Additionally, unlike the CSCG, the successor representation assumes full observability of the state, so it cannot be derived from partial or aliased observations.

We take the CSCG learned from the aliased 6×8 room in **Fig. 2a** and generate the SR from the CSCG transition matrix. Then we identify which clones correspond to which spatial locations by observing which clones activate in each location during inference. In **Supplementary Fig. 5a** we visualize the SR

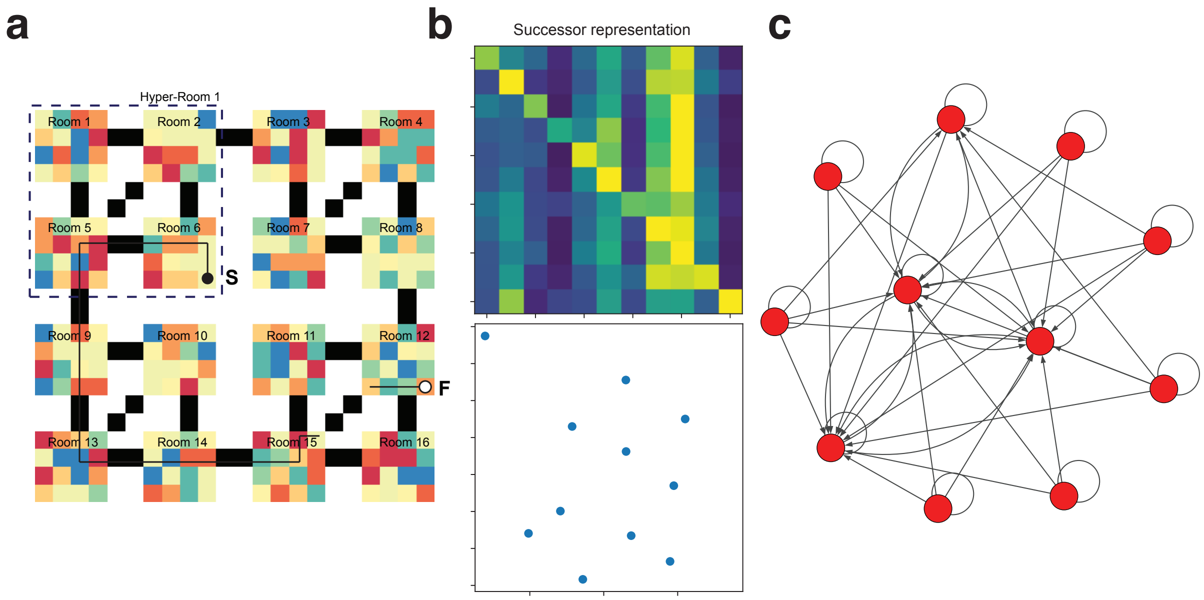

Supplementary Fig. 6: Successor representation for the hierarchical maze experiment. **(a)** Same maze as shown in **Fig. 7d**. **(b)** Successor Representation matrix and MDS. **(c)** Graph derived from Successor Representation Matrix.

954 for each hidden state. We also compute the eigenvectors of the matrix containing the SR of each state. We visualize these eigenvectors in **Supplementary Fig. 5b** and we also observe various grid patters of different scales, similarly to [60].

Supplementary Movie 1 Clone activations of an agent during navigation. The left panel shows the physical location of the agent and the local visual cue (color) available to it, whereas the right panel shows the inferred position in the agent's cognitive map (which has been learned from data). The agent only observes the current color (and not even its own actions). There are two patches (marked in black) that have identical colors, so at the beginning of exploration, the agent's belief in the cognitive map (right) is split between the two possible realities. As soon as the agent exits the duplicated patch, it can figure out its precise location and track it properly from that point on, as shown by the lack of ambiguity in the cognitive map when the agent returns to the repeated patch.

Supplementary Movie 2 Inferred cognitive map over learning iterations. The CSCG transition matrix is updated after each EM iteration, and the current state of the model is displayed as a cognitive map. To do this, the training data is decoded as a sequence of clones using Viterbi, and the resulting clone transitions are represented in a graph. The layout of the graph is obtained automatically using `python-igraph`.
