# Supplementary material for: Clone-structured graph representations enable flexible learning and vicarious evaluation of cognitive maps
Source: Nat Commun. 2021 Apr 22;12:2392. doi: 10.1038/s41467-021-22559-5 (PMC8062558; doi:10.1038/s41467-021-22559-5)
Supplement: Supplementary file 3 — Description of Additional Supplementary Files [file 41467_2021_22559_MOESM3_ESM.pdf]

Description of Additional Supplementary Files

File Name: Supplementary Movie 1
Description: : Clone activations of an agent during navigation. The left panel shows the physical location of the agent and the local visual cue (color) available to it, whereas the right panel shows the inferred position in the agent's cognitive map (which has been learned from data). The agent only observes the current color (and not even its own actions). There are two patches (marked in black) that have identical colors, so at the beginning of exploration, the agent's belief in the cognitive map (right) is split between the two possible realities. As soon as the agent exits the duplicated patch, it can figure out its precise location and track it properly from that point on, as shown by the lack of ambiguity in the cognitive map when the agent returns to the repeated patch.

File Name: Supplementary Movie 2
Description: Inferred cognitive map over learning iterations. The CSCG transition matrix is updated after each EM iteration, and the current state of the model is displayed as a cognitive map. To do this, the training data is decoded as a sequence of clones using Viterbi, and the resulting clone transitions are represented in a graph. The layout of the graph is obtained automatically using python-igraph.
