# Supplementary figures and images for: Clone-structured graph representations enable flexible learning and vicarious evaluation of cognitive maps
Source: Nat Commun. 2021 Apr 22;12:2392. doi: 10.1038/s41467-021-22559-5 (PMC8062558; doi:10.1038/s41467-021-22559-5)

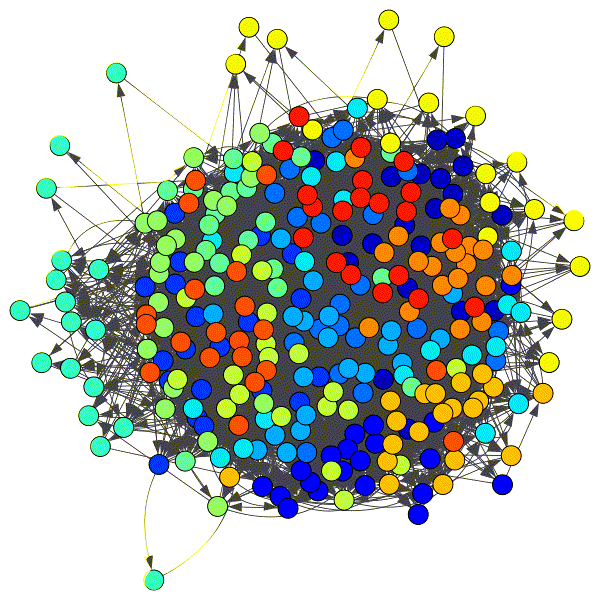

Supplement: Supplementary file 5 — Supplementary Movie 2 [file 41467_2021_22559_MOESM5_ESM.gif]
